# 'Choosing shoes': a preliminary study into the challenges facing clinicians in assessing footwear for rheumatoid patients

**DOI:** 10.1186/1757-1146-3-24

**Published:** 2010-10-19

**Authors:** Renee N Silvester, Anita E Williams, Nicola Dalbeth, Keith Rome

**Affiliations:** 1 AUT University, Health & Rehabilitation Research Institute, Auckland, New Zealand; 2 University of Salford, Directorate of Prosthetics, Orthotics and Podiatry, UK; 3 Auckland District Health Board, Auckland, New Zealand; 4 University of Auckland, Auckland, New Zealand

## Abstract

**Background:**

Footwear has been accepted as a therapeutic intervention for the foot affected by rheumatoid arthritis (RA). Evidence relating to the objective assessment of footwear in patients with RA is limited. The aims of this study were to identify current footwear styles, footwear characteristics, and factors that influence footwear choice experienced by patients with RA.

**Methods:**

Eighty patients with RA were recruited from rheumatology clinics during the summer months. Clinical characteristics, global function, and foot impairment and disability measures were recorded. Current footwear, footwear characteristics and the factors associated with choice of footwear were identified. Suitability of footwear was recorded using pre-determined criteria for assessing footwear type, based on a previous study of foot pain.

**Results:**

The patients had longstanding RA with moderate-to severe disability and impairment. The foot and ankle assessment demonstrated a low-arch profile with both forefoot and rearfoot structural deformities. Over 50% of shoes worn by patients were open-type footwear. More than 70% of patients' footwear was defined as being poor. Poor footwear characteristics such as heel rigidity and sole hardness were observed. Patients reported comfort (17%) and fit (14%) as important factors in choosing their own footwear. Only five percent (5%) of patients wore therapeutic footwear.

**Conclusions:**

The majority of patients with RA wear footwear that has been previously described as poor. Future work needs to aim to define and justify the specific features of footwear that may be of benefit to foot health for people with RA.

## Background

Therapeutic footwear that includes either retail, custom-made or off-the-shelf footwear is recommended for patients with diseases such as rheumatoid arthritis (RA) as a beneficial intervention for reducing foot pain, improving foot health, and increasing general mobility [1].

The foot is often the first area of the body to be systematically afflicted by RA [2-4]. Seventy-five percent (75%) of patients with RA report foot pain within four years of diagnosis, with the degree of disability progressing with the course of the disease [4]. Shi stated that virtually 100% of patients report foot problems within 10 years of disease onset [5]. The management goals for the RA foot are pain reduction, the preservation of foot function, and improved patient mobility [6].

A number of UK and European guidelines have recommended the use of therapeutic interventions for patients with RA [7]. One national guideline in the UK reported that therapeutic footwear should be available to all people with RA, if indicated [8]. In another UK study the authors reported that appropriate footwear for comfort, mobility and stability is well recognised in clinical practice but little available evidence for early RA [9]. In established RA extra-width off-the-shelf therapeutic shoes for prolonged use are indicated when other types of footwear have failed [10]. However, the level of supporting evidence is low, mainly at the 'good clinical practice' and 'expert opinion' agreement level [7].

A limitation to current recommended guidelines is an assessment tool to evaluate footwear specifically for RA. In a recent article pertaining to falls prevention in older adults the authors reported that In order for health care professionals to accurately and efficiently critique an individual's footwear and provide advice, a valid and reliable footwear assessment tool is required [11]. Such an assessment tool does not exist for footwear in patients with RA. The *Footwear Checklist *provides guidance to health professionals when assessing patients' footwear but is not specific to RA [12]. A *Footwear Assessment Tool *based upon postural stability and falls risk factors has also been reported [13]. The *Footwear Suitability Scale*, a measure of shoe fit for people with diabetes has also been reported [14].

To understand footwear characteristics determined by patients with RA, the aims of the study were to identify footwear style, footwear characteristics, and key factors influencing footwear choice using objective footwear assessment tools.

## Methods

### Patients

The study was conducted over 12 weeks between December 2009 and March 2010 (Southern Hemisphere summer). Sample size was determined by a fixed recruitment period for the study. Ethical approval was obtained from the Northern X Regional Ethics Committee, New Zealand. All patients gave informed consent to participate in the study. Patients with RA were recruited from rheumatology outpatient services based at Auckland District Health Board, Auckland, New Zealand. One examiner (RS) interviewed and assessed all patients. Patients were eligible if they had a diagnosis of RA according to the 1987 American Rheumatism Association revised criteria [15].

### Clinical characteristics

Age, ethnicity, gender, occupation, disease duration, Health Assessment Questionnaire [16] and current pharmacological management that include non-steroidal anti-inflammatory drugs (NSAIDs), methotrexate, other disease modifying anti-rheumatic drugs (DMARDs), prednisone and biologic therapies were recorded for each patient. Blood results (ESR and CRP) and the presence of radiographic erosions were also recorded.

### Foot and ankle assessment

Forefoot and rearfoot deformities were quantified using the Structural Index Score [17], which considers hallux valgus, metatarsophalangeal (MTP) subluxation, 5^th ^MTP exostosis, and claw/hammer toe deformities for the forefoot (range 0-12) and calcaneus valgus/varus angle, ankle range of motion and pes planus/cavus deformities for the rearfoot (range 0- 7). Foot type was assessed using the Foot Posture Index which is a validated method for quantifying standing foot posture [18]. The normal adult population mean Foot Posture Index score is +4, and scores above +4 suggest a flat-foot type. Hallux valgus [bunion] deformity was determined by the present or absence of a bunion.

### Disease measurement

Disease impact was measured using the Leeds Foot Impact Scale [19]. This self completed questionnaire comprises two subscales for impairment/footwear (LFISIF) and activity limitation/participation restriction (LFISAP). The former contains 21 items related to foot pain and joint stiffness as well as footwear related impairments and the latter contains 30 items related to activity limitation and participation restriction [19].Turner reported that a LFISIF >7 point and LFISAP >10 point as a high-to severe level of foot impairment and disability [20].

### Footwear assessment

An objective assessment of footwear was carried out by the examiner, to ascertain the type and appropriateness of the participant's current footwear. Menz and Sherrington [13] developed the seven item Footwear Assessment Form as a simple clinical tool to assess footwear characteristics related to postural stability and falls risk factors in older adults [11]. The assessment form allows clinicians to assess footwear style and footwear characteristics From a list of 16 styles of footwear, the examiner documented the style of shoe worn by the patient at the time of the assessment [13]. The footwear assessment tool has been reported to have good face validity and intra-tester reliability for use in older people [11,13].

Sandals are defined as shoes consisting of a sole fastened to the foot by thongs or straps. A mule shoe is a type of shoe that is backless and often closed-toed. The term jandals, used predominantly in New Zealand and the South Pacific (also known as flip-flops in the UK and US and thongs in Australia) are flat, backless, usually rubber sandal consisting of a flat sole held loosely on the foot by a Y-shaped strap that passes between the first and second toes and around either side of the foot.

Each shoe was assessed by the examiner for its construction and was based on the Footwear Assessment Form and included heel height (%); type of fixation (%); heel counter stiffness (%); midfoot sole sagittal rigidity (%) and forefoot sole flexion point at 1^st ^MPTJ (%) [11,13]. Categories for increased heel height were 0 to 2.5 cm, 2.6 to 5.0 cm, or > 5.0 cm) [11,13]. Measurement was recorded as the average of the height medially and laterally from the base of the heel to the centre of the heel-sole interface [11,13]. Types of fixation were categorised as none, laces, straps/buckles and Velcro [11,13]. Heel counter stiffness was categorised as none, minimal (> 45°), moderate (< 45°), or rigid (< 10°). To measure this, the heel counter was pressed with firm force approximately 20 mm from its base and the angular displacement estimated [11,13]. Midfoot sole sagittal stability was categorised as minimal (> 45°), moderate (< 45°), or rigid (< 10°). The examiner grasped both the rearfoot and forefoot components of the shoe and attempts were made to bend the shoe at the midfoot in the sagittal plane [11]. Forefoot sole flexion point was categorised as: at level of MPJs, proximal to MPJs, or distal to MPJs [11,13]. Tread pattern was divided into three items consisting of textured, partially worn or smooth [11,13].

**Figure 1:**
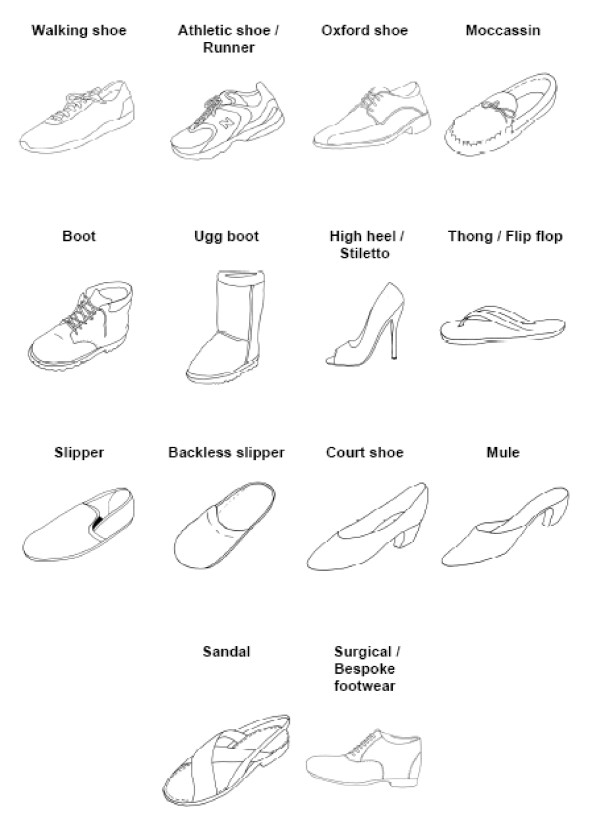
**Footwear types**. With permission from Barton CJ, Bonanno D, Menz HB. Development and evaluation of a tool for the assessment of footwear characteristics. J Foot Ankle Res 2009; 23: 10.

Based upon a previous study of patients with arthritic foot pain we classified current footwear into poor, average and good footwear [21]. The poor footwear group consisted of footwear that lack support and sound structure, including high-heeled shoes, court shoes, sandals, jandals, mules and moccasins. The average footwear group included shoes such as hard-or-rubber-soled shoes and work boots. The good footwear group consisted of athletic shoes, walking shoes, therapeutic footwear and Oxford-type shoes. A description of each shoe can be found in Figure 1.

Each patient was asked by the examiner to identify the most important features on a check-list. A list of factors included: comfort, style, fit, support, sole, weight, colour, uppers, fastenings, non-slippage, heel height and donning and doffing [22].The patient was given the opportunity to provide more than one response.

### Data Analysis

Data were analysed using SPSS 16.0 for Windows. Pharmacological management, gender, occupation, ethnicity and general footwear scores were described as n (percentages). All other demographic characteristics were described as the median (interquartile range - IQR). Secondary analysis evaluated the correlation between shoe type and foot function and structure using Pearson Chi-square.

## Results

### Participant Demographics & Disease Characteristics

Patients were predominantly middle-aged females with well established disease. The clinical characteristics are shown in Table 1.

**Table 1:** Demographic & Clinical Characteristics

Demographic Characteristics	Value
Median (IQR) Age (years)	60 (51-70)

Gender (F: M), n (%)	(4:1),
	Females: 64, (81%)
	Males: 15 (19%)

Ethnicity, n (%)	Caucasian, 50 (63%)
	Pacific Island, 8 (10%)
	Maori, 7 (9%)
	Asian, 9 (11%)
	Non-European Caucasian, 4 (5%)
	African, 2 (2%)

Median (IQR) disease duration (years)	11 (4-22)

Working: n (%)	30 (38%)

Not working/Beneficiary: n (%)	6 (7%)

Housewife/homemaker: n (%)	43 (54%)

**Clinical Characteristics**	

Median (IQR) HAQ Score (0-3)	0.7 (0.3, 1.35)

Radiographic erosions, n (%)	37 (51%)

History of Diabetes: n (%)	7 (9%)

**Pharmacological Management**	

NSAIDS: n (%)	25 (13%)

Methotrexate: n (%)	56 (29%)

Other DMARDS: n (%)	69 (35%)

Prednisone: n (%)	34 (17%)

Biologics: n (%)	11 (6%)

**Blood Investigations**	

Median (IQR) ESR (mm/hr)	17.0 (9, 45)

Median (IQR) CRP (mg/L)	4 (1.3; 13)

### Foot impairment

Patients in the current study had high-to severe (LFISIF >9 point, LFISAP >11 points) levels of foot impairment and disability on the LFIS subscales (Table 2). The forefoot structural index demonstrated severe structural problems but the rearfoot structural indices demonstrated moderate problems. The Foot Posture Index demonstrated the median [IQR] score of 8 [6,10]. Over 50% of patients were observed with hallux valgus (bunions).

**Table 2:** Relationship between shoe type (good, poor and average) and foot function and structure

Foot Function & Structure Characteristics	Median (IQR)
Forefoot Structural Index	7 (4,10)

Rearfoot Structural Index	4 (1,12)

Leeds Foot Impact Scale impairment/footwear	9 (6,12)

Leeds Foot Impact Scale activity limitation/participation restriction	11 (5,22)

Hallux Valgus: n (%)	51 (64%)

Foot Posture Index	8 (6,10)

### Footwear assessment

Patients were observed using open-toe footwear such as sandals (33%), jandals (10%), mules (6%) and moccasins (5%). Five percent (5%) of patients wore therapeutic footwear (Table 3). No subjects were found to be wearing 'average' footwear. Seventy percent (70%) of patients shoes were defined as 'poor' and 30% of patients were wearing good footwear.

**Table 3:** General Footwear Type

Footwear type	n (%)
Sandal	26 (33%)

Mule	5 (6%)

Jandals	8 (10%)

Walking Shoe	12 (15%)

Athletic Shoe	7 (9%)

Moccasin	4 (5%)

Therapeutic Footwear	4 (5%)

Boot	1 (1%)

High Heel	1 (1%)

Court Shoe	11 (14%)

Oxford Shoe	1 (1%)

Table 4 describes footwear characteristics. Over 80% of the current shoes had a heel-height between 0 and 2.cm. The majority of patient's footwear were observed with one fixation (46%), straps/buckles (35%) or laces (18%). A rigid heel counter stiffness was found in 40% of cases with over 38% of footwear unable to be assessed. Midfoot sole sagittal stability was found in 56% of shoes. A firm sole hardness was found to be in 56% of shoes with 35% of shoes were observed with soft sole hardness. Over 40% of shoes were found to partially worn, 41% with a textured surface and further 18% with a smooth surface. Over 85% demonstrated a forefoot sole flexion point at the 1^st ^MPTJ.

**Table 4:** Footwear Construction

Footwear Variable	n (%)
**Heel Height**	
0-2.5 cm	64 (80%)
2.6-5.0 cm	16 (20%)

**Fixation**	
One	36 (45%)
Laces	14 (18%)
Straps/Buckles	28 (35%)
Velcro	2 (3%)

**Heel Counter Stiffness**	
Not Available	30 (38%)
<45 degrees	18 (23%)
>45 degrees	32 (40%)

**Longitudinal Sole Rigidity**	
<45 degrees	34 (42%)
>45 degrees	46 (58%)

**Sole Flexion Point**	
At level of 1^st ^MPJT	68 (85%)
Before 1^st ^MPJT	12 (15%)

**Tread Pattern**	
Textured	33 (41%)
Smooth	14 (18%)
Partly worn	33 (41%)

**Sole Hardness**	
Soft	28 (35%)
Firm	40 (50%)
Hard	12 (15%)

Table 5 describes the factors patients perceived as important; most frequently identified factors were comfort (17%), fit (14%), support (9%), heel height (9%), don on/off (9%) and weight (7%).

**Table 5:** Factors relating to footwear choice

Factors	n (%)
Comfort	77 (17%)

Style	30 (7%)

Fit	60 (14%)

Support	39 (9%)

Sole	22 (5%)

Weight	32 (7%)

Colour	19 (4%)

Uppers	17 (4%)

Fastenings	38 (9%)

Non-slippage	32 (7%)

Heel-height	42 (9%)

Don on/off	37 (8%)

Secondary analysis demonstrated no significant correlation between footwear type (poor and good) and Leeds Foot Impact Scale, impairment domain (p = 0.243); Leeds Impact Scale, activity domain (p = 0.319); Foot Structural Index, rearfoot deformities (p = 0.592); Hallux valgus (p = 0.660) and Foot Posture Index (p = 0.724). However, a significant correlation was reported between footwear type and the Foot Structural Index, forefoot deformities (p = 0.008).

## Discussion

The aim of this study was to identify current footwear styles, footwear characteristics, and factors that influence footwear choice experienced by patients with RA. Overall, we found that moderate impairment and limited activity scores, consistent with significant foot disability. Foot deformities such as bunions were present in over 50% of patients with a low-arch profile. Forefoot structural deformities were high, suggesting that patients have problems in finding good footwear that accommodates structural changes in the forefoot and lesser extent in the rearfoot. Previous studies have also highlighted the problems of forefoot deformities in rheumatoid patients [23,24]. Helliwell further stated that patients with foot deformity find it increasingly difficult to buy footwear that can accommodate their foot shape as deformity progresses [23]. Difficulties in finding appropriate footwear due to forefoot structural deformities and the consequence wearing of inappropriate footwear can be a major contributing factor to foot impairment.

We found that the majority of patients were wearing court-shoes, sandals, moccasins, mules and jandals [jandals are specifically known to New Zealanders and other countries describe them as flip-flops or thongs]. One study reported that gait changes were observed in asymptomatic population with wearing flip-flops in and suggested that the shoe construction may contribute to lower limb leg pain and are counter-productive to alleviating pain [25]. The wearing of open-type footwear should be interpreted with caution. It is important to understand that open-type footwear, such as jandals and sandals are commonly worn in New Zealand, and the study was conducted during the summer. Future studies classifying footwear in patients with RA needs to take into cultural differences. Court-shoes were considered 'poor' due to lack of support mechanisms, cushioning and protection of toe regions possibly contributing to impairment and disability. Dixon argued that some of the foot deformities observed in RA, are the result of wearing of poor shoes, such as court shoes, although the authors do not substantiate this statement with any evidence [26].

The patients' choice of wearing athletic footwear in the current study reflects similar findings from a previous study that reports younger patients with RA (average age 58 years old) being prescribed athletic footwear as being 'acceptable', compared with off-the shelf orthopaedic footwear [27]. Helliwell also reported that many RA patients find athletic shoes the most comfortable option [23]. As the disease progresses the desire is to find wider fitting shoes to accommodate the broadening forefoot is needed and this is reflected in the high forefoot structural index score found in the current study. However, it is also reported that people with RA desire a choice in footwear according to their needs, particularly social needs and requirement in relation to seasonal variations [1]. Footwear such as therapeutic footwear or trainers may not meet those needs and this may be reflected in the current study in the higher use of sandals.

Despite the benefits of therapeutic footwear that have been previously reported [9,28-31], this type of footwear was not widely worn by patients in the current study. Additionally there are known factors relating to poor use of therapeutic footwear related to many factors that deem it unacceptable [1,32,33]. Williams identified therapeutic footwear as being the only intervention that we give that replaces something that is normally worn as an item of clothing and therefore reinforces the stigma of foot deformity and disability [1]. In addition to the body image issues Otter reported that that some patients discontinued using therapeutic footwear either because their foot symptoms had resolved or because they had foot surgery [32].

In the current study the participants reported that fit and comfort were important factors in choosing footwear, suggesting that patients prioritise fit due to their long-term disability. These findings are consistent with other reports [22]. Williams reported on the perception of features of five different pairs of off the shelf footwear [22]. Each patient was asked to examine the shoes and was then interviewed. Questions were asked about overall comfort, shoe style and fit. The results from interviews showed that in the rheumatoid group comfort was the primary factor followed by style and fit. Helliwell [23] has suggested that once the disease progresses the resulting pain and ensuing deformity makes obtaining comfortable footwear that fits a difficult task. Although patient's preference was for a 'poor' type of shoe, however, they reported them to be comfortable. This seems counter-intuitive and taken at face value perhaps there is a need to re-consider how footwear is classified. If 'poor' footwear is the most comfortable, much footwear advice given by health professionals may need re-evaluated and describing appropriate or good footwear should be incorporated into any short or long term management strategies.

In relation to the footwear characteristics we found that the majority of patients wore shoes that had an adequate heel height. On examining the fastening mechanism of the footwear, one strap/buckle was found in nearly 50% of shoes, possibly due to hand deformities that are often observed in patients with established RA may have contributed to the low number of shoes that used laces. Wear patterns on the footwear provided some indication in nearly 50% that they were partially worn. This aligns with comments made by the participants in relation to their choice of footwear for comfort and fit. Other footwear characteristics produced inconclusive results suggesting that the current assessment tool used in this study was not suitable for assessing footwear in patients with RA.

There are several limitations to this study that warrant discussion. The patients were recruited from one large city hospital during the summer months. The findings may not be a true representation of footwear styles in rural settings or during cooler seasons. A long term multicentre study is required to demonstrate geographical and seasonal differences in patients' preference of footwear style and type. The current study used a self-reported questionnaire to identify footwear style based upon postural stability and falls prevention. Future work needs to aim to define and justify the specific features of footwear that may be of benefit to foot health for people with RA in relation to their needs.

An important factor that was not included into the current study was direct or indirect costs. The wearing of poor shoes may have been due to financial constraints of purchasing 'good' footwear, i.e. direct costs to the patients. Furthermore, RA is a painful and distressing condition that can affect all ages and have a major impact on economically active adults, who may be forced to give up work either temporarily or permanently due to their condition, i.e. indirect costs. Therefore, clinicians and researchers should be aware of the direct and indirect costs to patients in obtaining 'good; footwear.

Secondary analysis demonstrated a significant correlation between footwear type and forefoot deformities using the Foot Structural Index. Tentatively, this suggests a link between presence of forefoot deformities and footwear. Since the majority of RA patients suffer from forefoot deformities, difficulties in finding 'good; footwear may exacerbate the already existing problems. The index is a qualitative tool providing an overall observation of forefoot and rearfoot deformities in quick and easy manner. However, the index has not been evaluated for its reliability. Helliwell [23] also reported that the index is limited to monitor subtle changes of foot deformity over time. Furthermore, the current study was cross-sectional. Future studies need to evaluate cause and effect before any definitive conclusions can be made looking at the relationship between footwear, foot type, foot pathologies and associated pain.

## Conclusions

This study has demonstrated that although fit and comfort were perceived by patients to be important factors in choosing footwear, current footwear choices are frequently inappropriate. Choices regarding footwear may reflect the difficulties patients with RA experience when obtaining footwear that meets their needs. This work has highlighted the need for good footwear and the need to improve both patient and practitioner knowledge of footwear.

## Competing interests

The authors declare that they have no competing interests.

## Authors' contributions

KR and ND conceived and designed the study. RS collected and inputted the data. KR and RS conducted the statistical analysis. KR and RS compiled the data and drafted the manuscript and RS, ND and AW contributed to the drafting of the manuscript. All authors read and approved the final manuscript.
